# Hemoperfusion and intravenous immunoglobulins for refractory gastrointestinal involvement in pediatric Henoch-Schönlein purpura: a single-center retrospective cohort study

**DOI:** 10.1186/s12887-022-03709-0

**Published:** 2022-12-02

**Authors:** Xiaolu Zhang, Ruochen Che, Haisheng Xu, Guixia Ding, Fei Zhao, Songming Huang, Aihua Zhang

**Affiliations:** 1 Department of Nephrology, Children’s Hospital of Nanjing Medical University, Nanjing, 210008 China; 2 Department of Emergency Medicine, Children’s Hospital of Nanjing Medical University, Nanjing, 210008 China; 3 Nanjing Key Laboratory of Pediatrics, Children’s Hospital of Nanjing Medical University, Nanjing, 210008 China

**Keywords:** Pediatrics, Henoch-Schönlein purpura, Refractory gastrointestinal symptoms, Hemoperfusion, Intravenous immunoglobulin G

## Abstract

**Background:**

Henoch-Schönlein purpura (HSP) with refractory gastrointestinal (GI) symptoms is always difficult to handle because of its resistance to supportive therapies and glucocorticoid. This study aimed to evaluate the efficacy of hemoperfusion (HP) and intravenous immunoglobulins (IVIG) therapies in this population.

**Methods:**

Sixty-four HSP patients with refractory GI involvement (R-GI group) and 64 cases with mild GI symptoms (control group) were retrospectively analyzed in our center from March 2016 to October 2019. In R-GI group, 42 cases (subgroup A) were treated with IVIG and steroid, 13 cases (subgroup B) used HP and steroid, 9 cases (subgroup C) executed a combination of IVIG, HP and steroid. Demographic characteristics, clinical features, laboratory indexes and treatment outcomes were recorded. *t*-test, One-way ANOVA, Mann-Whitney *U* test, and multivariate logistic regression were used in comparing differences among subgroups and predicting independent risk factors.

**Results:**

Compared with the control group, R-GI cases experienced higher risk of renal involvement (*P* = 0.000), more steroid exposure (*P* = 0.000), six times expenses (*P* = 0.000) and 2.3 times length of hospitalization (*P* = 0.000). The independent risk factors of R-GI group were elevated neutrophils (OR 1.250 [95% CI 1.130-1.383]) and the percentage of B lymphocytes (OR 1.100 [95% CI 1.026-1.179]) as well as decreased IgG (OR 0.847 [95% CI 0.732-0.98]). In R-GI group, increased age (OR 1.039 [95% CI 1.016-1.062]) and IgM (OR 5.994 [95% CI 1.403-27.611]) were verified to be risk factors of HSP nephritis. All three subgroups could alleviate the symptoms effectively. Compared with those in subgroup A, patients in subgroup B were elder (*P* = 0.004), had less relapse (*P* = 0.002), steroid exposure (*P* = 0.033) and expenses (*P* = 0.031), more significant decrease of WBC (*P* = 0.026) after treatment.

**Conclusion:**

The HSP with refractory GI involvement had much higher risk of medical burden and renal involvement. Both IVIG and HP therapies could ameliorate refractory GI symptoms efficiently. HP therapy tended to reduce the relapse, costs and steroid exposure in its audiences who were cooperated and with stable hemodynamics, while IVIG had better use in younger children.

## Background

Immunoglobulin A vasculitis (IgAV), also called Henoch-Schönlein purpura (HSP), is one of the most commonly seen vasculitis in children [1]. The reported incidence varied from 6 to 24 per 100,000 children younger than 17 years with a peak incidence at 4-6 years of age, depending on the ethnic background [2]. In Asia, the incidence could be as high as 56-70 per 100,000 children [3]. The major clinical manifestations are as followed: purpura, arthralgia/arthritis, abdominal pain and renal diseases. It is reported that 31-66.7% patients have the complaints of gastrointestinal (GI) symptoms, manifesting as paroxysmal umbilical pain, hematemesis, and hematochezia [4, 5]. Some patients can even progress to severe GI complications, such as intussusception, intestinal perforation and hemorrhagic enteritis [6]. Although HSP tends to be self-limiting, it still can develop some long-term complications. Approximately 20-60% of the HSP patients have kidney involvement, finally progressing to HSP nephritis (HSPN) [7]. Furthermore, permanent renal injury is reported in one-fifth of children with nephritic or nephrotic features [8]. A meta-analysis showed abdominal pain, gastrointestinal bleeding and severe bowel angina in HSP were among the potential risk factors of HSPN [9]. Indeed, more evidence supported that severe GI symptoms in early stage were related to the poor long-term renal prognosis of HSP patients [10–12], though the mechanism is not fully understood.

Unfortunately, unfavorable outcomes of GI involvement are also accompanied with limited therapy choices. To date, the mainstay of HSP management is generally supportive and symptomatic, like relief of pain, adequate hydration and full rest. Glucocorticoids are the most commonly used medicines when severe abdominal pain and joint symptoms exist. Evidence showed that early use of glucocorticoid benefits for controlling GI symptoms, but it does not appear to otherwise impact the clinical course [13, 14]. Moreover, there’s still a part of HSP patients with refractory GI symptoms failed to achieve remission even by using glucocorticoid [15]. Immunosuppressants, immunoglobulin G, hemoperfusion and other second-line therapies would then be considered. There is currently no consensus for the treatment of these patients. In another way, there’s few guidelines or high-quality evidence on the management of steroid-resistant or dependent HSP with refractory GI symptoms, which were only mentioned in five series of small-sized studies [16–20]. In 2013, the Chinese guidelines put forward that intravenous immunoglobulin G (IVIG) and hemoperfusion (HP) might have beneficial effects on severe HSP during the acute phase with weak evidence support (Evidence grade V/E) [10]. Actually, based on several reports, compared with steroid and immunosuppressive agents, IVIG and HP are free of long-term side effects, which are preferred in patients with steroid-resistant or dependent GI complications in some centers [20, 21]. Furthermore, these therapies may especially suitable for those who are unable to intake medicine and food orally because of severe GI symptoms.

Given the obscure evidence of both IVIG and HP therapies, the aim of the study was not only to conclude the clinical features and outcomes of a large group of pediatric HSP patients with refractory GI symptoms but also compare the efficacy of IVIG and HP therapies in this population.

## Methods

### Ethical approval

This study was conducted in accordance with the principles of the Declaration of Helsinki. Study approval was obtained from the institutional review board of Children’s Hospital of Nanjing Medical University. The data were collected retrospectively, and without involving patients’ privacy and biological samples. Therefore, the institutional review board of Children’s Hospital of Nanjing Medical University approved the informed consent waiver.

### Diagnostic criteria and definition

The diagnostic criteria for HSP was based on the 2005 European League Against Rheumatism (EULAR) and the Pediatric Rheumatology European Society (PRES), which was subsequently validated in conjunction with the Pediatric Rheumatology International Trials Organization (PRINTO) [22, 23]. Detailed criteria includes the presence of palpable purpura or petechiae without thrombocytopenia or coagulopathy, with one or more of the following symptoms: (1) arthritis/arthralgia; (2) abdominal pain; (3) renal involvement; (4) leukocytoclastic vasculitis or proliferative glomerulonephritis, with predominant IgA deposition. Refractory GI involvement was defined as the persistent GI symptoms that did not remit after 3 days of glucocorticoid treatment (prednisone 1-2 mg/kg/d orally, maximum dose of 60 to 80 mg per day or equivalent doses of parenteral dexamethasone) or dependent (relapsing twice when glucocorticoid was tapered) [18, 24]. Transient proteinuria/hematuria indicated that episodes of hematuria (urinary red blood cell (RBC) > 5/high-power field (HPF)) and/or proteinuria (urinary protein ≥100 mg/l or ≥ 150 mg/d) but resolved spontaneously in a week. Renal involvement in HSP refers to persistent hematuria and/or proteinuria (urinary protein ≥100 mg/l or ≥ 150 mg/d) and/or elevated serum creatinine (Scr), which happened within the first 6 months, and the renal biopsy results were interpreted based on International Study of Kidney Disease in Children (ISKDC) classifications and nephrology group, Chinese branch of pediatrics, Chinese medical association in 2016 [25–27]. GI Remission was defined as free of GI symptoms.

### Participants and design

Medical records of children aged ≤18 years with the diagnosis of IgA vasculitis in the division of Pediatric Nephrology, Children’s Hospital of Nanjing Medical University (Nanjing, China) from March 2016 to October 2019 were retrospectively reviewed for demographic data, clinical manifestations and laboratory tests. A total of 64 HSP patients with refractory GI involvement were allocated into the R-GI group. HSP Patients who had GI symptoms but were relieved by symptomatic treatment or glucocorticoid rapidly, were included in the control group. To pair up, 64 cases were selected utilizing stratified random sampling by age and gender from 1015 mild cases.

The clinical features, clinical data, including baseline information (age, sex, bodyweight), laboratory findings (blood cell count, urinalysis, stool test, CRP, procalcitonin (PCT), ESR, serum globulin, immunoglobulin A (IgA), immunoglobulin G (IgG), immunoglobulin M (IgM), the percentage of B lymphocyte in the whole lymphocytes (BLY%), CD4+/CD8+, serum creatinine (Scr), blood urea nitrogen (BUN), treatment expenses, the length of hospitalization (LOH), and renal outcomes were collected. Laboratory results of pre-treatment were assessed at the time of diagnosis, and post-treatment indexes were collected after completing therapies and remission.

### Treatment protocols

The control cases received the symptomatic treatment or glucocorticoid (prednisone 1-2 mg/kg/d orally, maximum dose of 60 to 80 mg per day or equivalent doses of parenteral dexamethasone) to relieve the symptoms. There were three subgroups in the R-GI group, the observation endpoint was the complete relief of the symptoms. Group A received one dose of IVIG (2 g/kg) combined with glucocorticoid (dose mentioned above). Group B received HP every other day for 5-8 times as well as glucocorticoid. If neither group A nor B could achieve the remission, they were allocated to group C and accepted either HP or IVIG therapy again. The decision of choosing IVIG or HP as the secondary therapy was mainly made by senior pediatricians, who usually took age and weight into account. Additionally, the guardian’s willingness would be also taken into consideration. Generally speaking, hemoperfusion was utilized in patients who were elder and complied with invasive procedure. Others would choose the IVIG first.

### Hemoperfusion

As an extracorporeal blood purification modality, hemoperfusion selectively eliminates abnormal cells, components, and cytokines in the blood due to specific disease states [28]. As previously reported, hemoperfusion can not only assist in remitting HSP children’s symptoms at the acute phase by reducing the inflammation and oxidative stress, but also contribute to treating severe abdominal and kidney complications of HSP [21, 29, 30]. And the treatment of hemoperfusion combined with corticosteroid was found to be more effective by eliminating serum immune mediators than corticosteroid alone in HSPN [31]. Patients were undergone internal jugular vein catheterization with local anesthesia. Hemoperfusion was conducted using dialysis blood lines and a dialysis machine apparatus containing a blood pump and pressure gauges. The HA 130/280 type hemoperfusion cartridges (Jafron, China) were utilized. The cartridges contain highly biocompatible sorbents and neutro-macroporous resin made of styrene-divinylbenzene copolymer that absorb harmful ingredients rapidly. In our center, we used low-molecular-weight heparin as the anticoagulation. The hemoperfusion is usually performed for approximately four hours.

### Statistical analysis

All data were statistically analyzed by SPSS 25.0 software. Continuous variables were reported as mean ± standard deviation, while categorical variables were presented by numbers (percentage). Differences between groups were compared by *t*-test, One-way ANOVA or Mann-Whitney *U* test, paired *t*-test or Wilcoxon paired signed-rank test were used in comparing differences between paired groups. χ^2^ or Fisher exact tests were used in categorical variables. Univariate and multivariate logistic regression analysis were performed to detect independent risk factors. And *P* value < 0.05 was considered as statistical significance.

## Results

### Demographic and clinical characteristics of control group and R-GI group

All demographic and clinical characteristics of patients were shown in Table 1. The control group consisted of 35 boys and 29 girls, with a mean onset age of 7.1 (4.3, 9.9) years. 44 boys and 20 girls with a mean age of 7.2 (4.2, 10.3) years were recruited in R-GI group. There were no significant differences between the control group and R-GI group in terms of age, sex, bodyweight.

**Table 1:** Comparison of clinical data between control group and R-GI group

Variables	Control group (*n* = 64)	R-GI group (*n* = 64)	*P*
Gender(M/F)	35/29	44/20	0.103
Age (years)	7.09 ± 2.77	7.20 ± 3.05	0.820
Weight (kg)	26.53 ± 11.13	26.90 ± 13.42	0.864
WBC (10^9/L)	12.30 ± 3.94	16.53 ± 7.29	**0.000**
Neut (10^9/L)	7.90 ± 3.88	14.50 ± 7.75	**0.000**
LY (10^9/L)	3.68 ± 1.50	3.08 ± 1.72	**0.039**
NLR	2.68 ± 2.11	6.53 ± 5.70	**0.000**
PLR	115.43 ± 60.73	157.88 ± 102.23	**0.005**
CRP (normal)	62(96.88%)	40(62.50%)	**0.000**
ESR (mm/h)	16.02 ± 14.32	10.70 ± 9.44	**0.048**
IgG(g/L)	11.06 ± 3.85	9.07 ± 3.45	**0.003**
IgA(g/L)	2.51 ± 1.45	2.09 ± 0.97	0.062
IgM(g/L)	1.25 ± 0.45	1.08 ± 0.45	**0.039**
Globulin(g/L)	26.00 ± 6.18	24.33 ± 4.51	0.082
BLY (%)	20.80 ± 5.99	26.92 ± 8.84	**0.000**
CD4+/CD8+	1.06 ± 0.40	1.21 ± 0.54	0.078
U-protein (negative)	63(98.44%)	38(59.38%)	**0.000**
U-RBC (/ul)	18.13 ± 70.92	126.62 ± 358.19	**0.020**
Joint involvement	16(25.00%)	34(53.13%)	**0.001**
Renal involvement	8(12.50%)	26(40.63%)	**0.000**
HSPN	5(7.81%)	19(29.69%)	**0.002**
GI bleeding	2(3.13%)	44(68.75%)	**0.000**
WCCDS (mg/kg)	19.09 ± 9.60	47.15 ± 16.71	**0.000**
TE (RMB)	2068.13 ± 1298.23	13,213.35 ± 6574.12	**0.000**
LH (days)	9.33 ± 3.99	21.42 ± 8.52	**0.000**

*Legends*: The table outlined the baseline clinical characteristics of control group and refractory GI group respectively, and showed the *P* value (P) of comparison in these characteristics between two groups. Data were expressed as n(%), mean ± standard deviation

*WBC* white blood cell counts, *Neut* Neutrophils counts, *LY* Lymphocyte counts, *CRP* C-reactive protein, *NLR* neutrophil/lymphocyte ratio, *PLR* platelet/lymphocyte ratio, *ESR* erythrocyte sedimentation rate, *IgG* serum immunoglobulin G, *IgA* serum immunoglobulin A, *IgM* serum immunoglobulin M, *BLY* B lymphocytes, *U-protein* urinary protein, *24 h u-protein* 24 hours urinary protein, *U-RBC* urinary RBC counts, *HSPN* Henoch-Schönlein purpura nephritis, *GI bleeding* gastrointestinal bleeding, *WCCDS* weight-corrected cumulative dose of steroid (coverted into prednisone dosage), *TE* treatment expenses, *LH* length of hospitalization

As mentioned before, R-GI group was divided into three subgroups. There were 42 R-GI cases (27 boys and 15 girls, mean onset age of 6.5 years) in the group A, 13 R-GI cases (11 boys and 2 girls, mean onset age of 9.2 years) in the group B, and 9 R-GI cases (6 boys and 3 girls, mean onset age of 7.6 years) in the group C. Patients of group A were the youngest and had the lowest weight, experiencing the shortest hospital stay (*P* = 0.016, 0.001, and 0.005, respectively), while there was no statistical difference of gender among three subgroups, shown in Table 2.

**Table 2:** Comparison of clinical data among three subgroups

Characteristics	Group A (IVIG)(*n* = 42)	Group B (HP)(*n* = 13)	Group C (IVIG+HP)(*n* = 9)	P_*_	P_**_	P_***_	*P*_§_
Age (years)	6.50 ± 2.63	9.22 ± 3.40	7.57 ± 3.33	**0.004**	0.193	0.322	**0.016**
Gender(M/F)	27/15	11/2	6/3	0.195	0.609	1.000	0.387
Weight (kg)	23.18 ± 8.93	38.92 ± 18.31	26.92 ± 14.12	**0.000**	**0.025**	0.402	**0.001**
NLR	5.96 ± 6.07	8.47 ± 4.98	6.38 ± 4.65	0.171	0.401	0.842	0.387
PLR	155.24 ± 117.84	178.71 ± 69.10	140.09 ± 53.56	0.476	0.392	0.691	0.664
LH (days)	18.98 ± 7.58	26.0 ± 10.11	26.22 ± 5.70	**0.007**	0.949	**0.016**	**0.005**
TE (RMB)	10,316.0 ± 5445.91	7015.08 ± 1629.44	18,976.00 ± 3726.21	**0.031**	**0.000**	**0.000**	**0.000**
WCCDS (mg/kg)	48.79 ± 16.82	37.61 ± 13.73	53.26 ± 16.15	**0.033**	**0.029**	0.454	0.051
DARGIS	10.79 ± 6.15	11.62 ± 5.97	13.89 ± 4.65	0.661	0.381	0.160	0.365
WL rate	0.075 ± 0.048	0.058 ± 0.031	0.069 ± 0.034	0.228	0.550	0.733	0.478
GI bleeding	27(64.29%)	9(69.23%)	8(88.89%)	0.745	0.291	0.153	0.357
Arthralgia	23(54.76%)	4(30.77%)	7(77.78%)	0.205	0.080	0.277	0.092
RI	15(35.71%)	6(46.15%)	5(38.46%)	0.529	1.000	0.454	0.498
HSPN	11(26.19%)	5(38.46%)	3(33.33%)	1.000	0.616	0.681	0.779

*Legends*: The table represented the comparison of baseline and clinical characteristics in three subgroups (groupA, B and C) of severe cases. P_*_ value of comparison between group A and B, P_**_ value of comparison between group B and C, P_***_ value of comparison between group A and C, *P*_§_ value of comparison between group A, B and C. Data are expressed as n(%), mean ± standard deviation

*NLR* neutrophil/lymphocyte ratio, *PLR* platelet/lymphocyte ratio, *LH* length of hospitalization, *TE* treatment expenses, *WCCDS* weight-corrected cumulative dose of steroid (coverted into prednisone dosage), *DARGIS* duration of achieving remission of gastrointestinal symptoms, *WL* weight loss, *RI* renal involvement, *HSPN* Henoch-Schönlein purpura nephritis

In the group A, 15 of 42 (35.71%) had transient proteinuria/hematuria, and HSPN was observed in 11 of 42 (26.19%) cases among whom 3 were unclassified and 8 underwent renal biopsy with 6 were grade IIIa HSPN and 2 were grade IIa HSPN. In the group B, 6 of 13 (46.15%) had transient proteinuria/hematuria while 5 of 13 (38.46%) finally progressed to HSPN of which 2 were unclassified, 2 were grade IIIa HSPN and 1 was grade IIb HSPN. In the 9 cases of group C, transient proteinuria/hematuria was exhibited in 5 cases, and 3 cases were involved in HSPN of which 1 was grade IIIa and 2 were unclassified.

### More adverse events and heavier socioeconomic burden were observed in R-GI group

Joint involvement and GI bleeding were more frequent in the R-GI group than those in the control group (53.13% vs. 25.00%, *P* = 0.001 and 68.75% vs. 3.13%, *P* = 0.000). Additionally, more weight-corrected cumulative dose of steroid (converted into prednisone dosage) was observed in the R-GI group compared with the control group (47.15 ± 16.71 vs. 19.09 ± 9.60, *P* = 0.000). The treatment expenses and LOH were also significantly higher in the R-GI group (*P* = 0.000 and 0.000, respectively).

Before the treatment, the level of WBC, lymphocytes (LY), neutrophils count, BLY%, NLR, PLR were significantly increased in R-GI group (*P* = 0.000, 0.039, 0.000, 0.000, 0.000 and 0.005, respectively), as well as the percentage of patients with normal CRP tended to be higher in control group (*P* = 0.000). IgG and IgM level were obviously decreased (*P* = 0.003 and 0.039, respectively) in R-GI group. Although serum globulin and IgA in R-GI group were lower, there were no differences between the control group and R-GI group.

Transient proteinuria/hematuria and HSPN were observed more frequently in R-GI group than that in control group (40.63% vs.12.50%, *P* = 0.000 and 29.69% vs. 7.81%, *P* = 0.002). In R-GI group, 26 cases had transient proteinuria/hematuria, 19 cases finally progressed to HSPN of which 7 were unclassified, 12 underwent renal biopsy with 9 were grade IIIa HSPN and 3 were grade II HSPN (2IIa and 1IIb), based on International Study of Kidney Disease in Children (ISKDC) classification [32], all were shown in Table 1.

The elevated neutrophils count (OR 1.250, 95% CI 1.130-1.383, *P* = 0.000) and BLY% (OR 1.100, 95% CI 1.026-1.179, *P* = 0.007), decreased IgG (OR 0.847, 95% CI 0.732-0.98, *P* = 0.026) level were independent risk factors of refractory GI involvement in HSP (Table 3).

**Table 3:** Logistic regression analysis of independent risk factors of refractory gastrointestinal involvement and HSPN in HSP

Variables	Univariable analysis			Multivariable analysis		
	OR	95%CI	*P*	OR	95%CI	*P*
**Risk factors of refractory GI involvement in HSP**						
Neut	1.259	1.126-1.408	**0.000**	1.250	1.130-1.383	**0.000**
IgG	0.836	0.724-0.964	**0.014**	0.847	0.732-0.981	**0.026**
IgM	0.285	0.082-0.988	**0.048**	0.434	0.160-1.180	0.102
BLY	1.109	1.025-1.200	**0.010**	1.100	1.026-1.179	**0.007**
**Risk factors of HSPN in patients with GI involvement**						
Age	1.026	1.009-1.043	**0.002**	1.026	1.009-1.043	**0.002**
IgM	3.165	1.001-10.005	**0.050**	3.165	1.001-10.005	**0.050**
GI bleeding	4.722	1.420-15.704	**0.011**	4.722	1.420-15.704	**0.011**
**Risk factors of HSPN in the R-GI group**						
Age	1.039	1.016-1.062	**0.001**	1.039	1.016-1.062	**0.001**
IgM	5.994	1.403-27.611	**0.016**	5.994	1.403-27.611	**0.016**

*Legends*: The table was consisted of three independent risk factors of refractory GI involvement in HSP, two risk factors associated with HSPN in HSP patients with GI involvement and two risk factors of HSPN in HSP patients with refractory GI involvement by multivariable logistic regression analysis

*Neut* Neutrophil counts, *IgG* serum immunoglobulin G, *IgM* serum immunoglobulin M, *BLY* B lymphocytes, *GI bleeding* gastrointestinal bleeding, *HSPN* Henoch-Schönlein purpura nephritis, *R-GI* refractory gastrointestinal

### Independent risk factors of progressing to HSPN in R-GI group

As shown above, patients in R-GI group were more susceptible with poor renal outcomes. Therefore, we believe it is more valuable to analyze the possible risk factors of HSPN in this special group. The multivariable logistic regression analysis showed that the increased age (OR 1.039, 95% CI 1.016-1.062, *P* = 0.001) and IgM (OR 5.994, 95% CI 1.403-27.611, *P* = 0.016) were verified to be the independent risk factors for progressing to HSPN in the population of R-GI group, all were shown in Table 3.

### Full remission, less steroid exposure and expenses were observed in the group of HP therapy

Although group B (HP therapy) had longer hospitalization than group A (IVIG therapy) (*P* = 0.007), the length of complete remission of GI symptoms showed no differences among three groups. The patients in group B all achieved full remission after the therapy in the first time while 17.6% patients failed to achieve the remission after IVIG therapy. Additionally, compared with group A and C, group B had the lowest weight-corrected cumulative dose of steroid (*P* = 0.033 and 0.029, respectively), only 70-77% of other two groups. And the medical expense of group A and C was approximately 1.5 and 2.7 times of group B, respectively (*P* = 0.000) (shown in Table 2).

Furthermore, the WBC had the most decrease after HP therapy among three subgroups (*P* = 0.026), and an obvious reduction of WBC and CRP was observed in group A compared with group C (*P* = 0.000 and 0.028, respectively), illustrated in Table 4. While other indexes such as urinary RBC counts, urinary protein and BUN showed no significant differences between pre- and post-treatment. Similarly, neither IVIG nor HP did not significantly influence the short-term and long-term renal outcomes, all were shown in Table 2 and 4. Because some specific test results, like immunological panels, PCT and ESR, were absent after the treatment, it was difficult for analyzing the change of IgA, IgG, IgM, BLY%, CD4/CD8, PCT and ESR between pre- and post- treatment in three subgroups.

**Table 4:** Changes of laboratory indexes between pre- and post-treatment in three subgroups

Indexes	GroupA		*P*	GroupB		*P*	GroupC		*P*
	Pre-	Post-		Pre-	Post-		Pre-	Post-	
WBC(10^9/L)	16.31 ± 7.16	11.53 ± 4.75	**0.000**	21.08 ± 8.81	13.51 ± 3.40	**0.026**	12.96 ± 3.64	13.10 ± 4.07	0.935
Neut (10^9/L)	14.58 ± 8.06	10.86 ± 13.20	0.207	16.25 ± 7.95	8.13 ± 2.44	0.055	16.91 ± 7.07	8.80 ± 2.75	0.084
CRP (normal)	30(71.43%)	32(76.19%)	**0.028**	6(46.16%)	11(84.62%)	0.069	4(44.44%)	7(77.78%)	0.430
Globulin(g/L)	24.35 ± 4.42	32.90 ± 8.05	**0.001**	25.08 ± 3.42	24.63 ± 7.00	0.852	24.87 ± 7.21	24.57 ± 5.93	0.906
U-protein(N)	26(61.90%)	35(83.33%)	0.172	9(69.23%)	7(53.85%)	0.419	3(33.33%)	6(66.67%)	0.720
U-RBC (/ul)	72.49 ± 282.06	222.52 ± 789.68	0.196	380.36 ± 901.07	408.93 ± 900.43	0.338	160.1 ± 191.62	252.05 ± 443.11	0.571
Scr (mmol/L)	34.72 ± 12.48	32.96 ± 14.89	0.457	44.62 ± 10.63	44.63 ± 14.42	0.994	35.60 ± 13.28	31.07 ± 11.69	**0.001**
BUN (mmol/L)	4.55 ± 2.05	4.61 ± 1.57	0.895	5.19 ± 2.14	5.23 ± 1.62	0.958	5.31 ± 1.91	3.55 ± 1.00	0.057

*Legends*: The table represents the *P* value(P) of the comparison of pre- and post-treatment laboratory findings in three subgroups (group A, B and C) in R-GI group. Data are expressed as n(%), mean ± standard deviation

*WBC* white blood cell counts, *Neut* Neutrophil counts, *CRP* C-reactive protein, *U-protein* urinary protein, *U-RBC* urinary RBC counts, *Scr* serum creatinine, *BUN* blood urea nitrogen, *Pre-* Pre-treatment, *Post-* Post-treatment

## Discussion

GI involvement, one of the most complicated symptoms in HSP, ranging from mild abdominal pain, vomiting to severe GI hemorrhage, intussusception, intestinal perforation and obstruction [33], commonly occurs in children and young adults [34]. In our work, approximately 5.30% HSP patients would develop refractory GI symptoms. Although the percentage is not so high, this group was in significantly higher risk of GI bleeding and renal involvement. Actually, various evidence supported that abdominal pain and gastrointestinal bleeding were the independent risk factors for HSPN [35–37]. Moreover, the study showed that refractory GI involvement in HSP brought heavy burdens to the families, as the treatment expenses increased by 6.4 times, the length of hospitalization extended by more than 12 days, and the incidence of poor long-term renal outcomes increased by 3.8 times, compared with mild GI symptoms cases. Therefore, special attention should be paid to those patients.

Unlike the previous studies, we analyzed the independent risk factors of developing HSP with refractory GI symptoms, rather than HSP alone. We found that severe cases were observed in HSP patients with GI symptoms who had elevated neutrophils and BLY% or decreased IgG level, a similar trend was found in one previous study that low level of IgG was associated with GI involvement [38]. Currently, persistent purpura, severe abdominal symptoms and an age of over four years were verified as the risk factors of HSPN [37, 39]. A few cohort studies verified that high urinary IgA, IgG, IgM were more indicative of renal involvement in IgAV patients [40, 41]. But there was no knowledge of risk factors associated with renal involvement in high-risk population of HSPN who presented with severe GI symptoms. In our R-GI group, increased age and IgM level were the independent risk factors of HSPN progression. Based on the previous study, it similarly revealed that IgM deposition in the kidney led by high circulating IgM was related to renal involvement in R-GI cases [40].

At present, the managements of refractory HSP patients remain variable, including steroid, immunosuppressants, IVIG and HP [12, 15]. The efficacy of steroid or various immunosuppressive agents have been explored worldwide [12, 15, 42], but IVIG or HP therapy was rarely mentioned. In 2013, Chinese guidelines put forward the role of IVIG and HP in relieving severe GI symptoms of HSP [10], mainly utilized in patients with steroid-resistant or -dependent severe complications, including necrotizing dermatitis, severe GI and neurological symptoms. To our knowledge, it is the first retrospective comparison of IVIG and HP therapies in HSP with refractory GI symptoms. Our results exhibited that GI symptoms were all relieved after five times of HP on average, and 82.35% were relieved after one dose of IVIG. Rises in WBC paralleled the increase risk for HSP [43–45], while both IVIG and HP were verified effective in reducing WBC significantly. However, the absence of immunological panels after treatment made it difficult for comparing immunological indexes, hence further attempt was required for comparing the immunological changes of two therapies.

On the one hand, IVIG is a kind of blood products which mixed by normal human immunoglobulin from several thousand healthy donors, containing most IgG and a small amount of IgA and IgM. A high dose (1-2 g/kg) IVIG was applied in autoimmune and inflammatory diseases, of which the mechanism is complex [46]. IVIG might act as an immune-modulator regulating various cells of the innate and adaptive immune compartments. The IgG-Fc fragment interacts with the Fcγ receptor on target cells, which appears to play the key role in anti-inflammation [47]. In children, IVIG treatment has been the standard first-line therapy in Kawasaki disease, idiopathic thrombocytopenic purpura and Guillain-Barré syndrome. But the data on the use of IVIG in the other types of auto-immune diseases are limited. For HSP, there were only scattered reports utilizing IVIG as an alternative when other treatment approaches failed. Mauro et al. recently reported a case of HSP presenting with severe skin lesions that did not respond to standard therapy with corticosteroids. The case accepted IVIG infusion (2 g/kg) and showed significant improvement in her skin lesions [48]. A French retrospective study investigated 9 patients with severe GI involvement who underwent IVIG therapy. The results showed a well response to the treatment [20]. Other case reports also supported that IVIG provides an effective alternative to corticosteroids in cases with severe GI symptoms [49, 50]. On the other hand, HP has been regarded as a new treatment in severe HSP, which eliminates the immune complexes and inflammatory factors in the blood through extracorporeal circulation [29, 30, 51]. Zhu et al. explored thirty HSP children with GI symptoms accepting the HP therapy and found that compared with corticosteroids, HP could effectively remove IL-6, TNF-α, MDA in the acute phase, decreasing glucocorticoid dosage and the rate of renal involvement [21]. Ma et al. also mentioned that HP could protect renal function of HSP children [29]. But in our study, we only compared the renal outcome in three subgroups of RGI patients. So, we did not observe the renal outcome differences in both IVIG and HP therapies. The HP therapy has been used cautiously in the past years, especially due to the increased risk of bleeding. In this study, we did not observe any reported side effects like headache, hypovolemia, anaphylaxis, leukopenia [20]. In our center, IVIG tended to be used in those with lower weight while HP was utilized in heavier patients. It is known that the expense of IVIG rises with the increase of weight while HP has its disadvantage in maintaining hemodynamic stabilization of younger children. Therefore, based on our experiences, proper weight is an ideal threshold for determining whether IVIG or HP is more suitable. Compared with single dose of IVIG therapy, the LOH in patients undergone HP was significantly prolonged, possibly because of longer period required for glucocorticoid withdrawal due to the relative larger total dosage and repeated HP therapies. However, it is interesting to find that the duration of achieving complete remission of GI symptoms had no differences between IVIG and HP therapies, but repeated HP was more beneficial for eliminating the inflammation.

There are several limitations in our study. Firstly, it is a retrospective design which grouping was based on the real world study rather than randomized controlled trials. Thus, the selection bias was hard to be avoided. Additionally, more comparable pre- and post-treatment laboratory indexes should be recorded so that a clearer picture of immunological change would be obtained. Finally based on our single-center experience, we lack the group of immunosuppressants. Therefore, it is difficult to evaluate the efficacy of immunosuppressants in treating HSP with refractory GI symptoms.

## Conclusion

This study retrospectively analyzed that HSP patients with refractory GI symptoms had more active inflammation status, longer hospital stays, higher treatment expenses and more steroid exposure. Children suffered from HSP with GI symptoms who had elevated neutrophils count, BLY% and reduced IgG were prone to progress to refractory GI involvements. Additionally, elder age and elevated IgM level were independent risk factor of HSPN in R-GI group.

Both IVIG and HP therapies could reduce WBC and ameliorate refractory GI symptoms efficiently. HP therapy tended to reduce the relapse, costs and steroid exposure in its audiences who were cooperated and with stable hemodynamics, while IVIG had better use in younger children. Renal outcomes were not influenced by the early start of either therapy. This study provides therapeutic options of refractory GI involvement in HSP as well as the evidence for identifying HSP patients with refractory GI involvement who were susceptible with poor renal outcomes. Additionally, a double-blind controlled study would be done in our further work, which includes a group treated with immunosuppressant to make a more comprehensive comparison.

## Data Availability

The data that support the findings of this study are available from Children’s Hospital of Nanjing Medical University but restrictions apply to the availability of these data, which were used under license for the current study, and so are not publicly available. Data are however available from the corresponding author Aihua Zhang (E-mail: zhaihua@njmu.edu.cn, telephone: + 86-25-8311-7504) upon reasonable request and with permission of Children’s Hospital of Nanjing Medical University.

## Abbreviations

BLY: B lymphocytes
BUN: blood urea nitrogen
CRP: C-reactive protein
DARGIS: duration of achieving remission of gastrointestinal symptoms
ESR: erythrocyte sedimentation rate
GI: gastrointestinal
HP: hemoperfusion
HSP: Henoch-Schönlein purpura
HSPN: Henoch-Schönlein purpura nephritis
IgA: immunoglobulin A
IgAV: Immunoglobulin A vasculitis
IgG: immunoglobulin G
IgM: immunoglobulin M
IVIG: intravenous immunoglobulins
LH: length of hospitalization
LOH: length of hospitalization
LY: Lymphocyte counts
Neut: Neutrophils counts
NLR: neutrophil/lymphocyte ratio
PCT: procalcitonin
PLR: platelet/lymphocyte ratio
RBC: red blood cell counts
RGI: refractory gastrointestinal
RI: renal involvement
Scr: serum creatinine
TE: treatment expenses
U-protein: urinary protein
U-RBC: urinary RBC counts
WBC: white blood cell counts
WCCDS: weight-corrected cumulative dose of steroid (coverted into prednisone dosage)
WL: weight loss
